# Conjunctival nevi: clinical and histopathologic features in a Saudi population

**DOI:** 10.4103/0256-4947.65265

**Published:** 2010

**Authors:** Hind M. Alkatan, Khalid M. Al-Arfaj, Azza Maktabi

**Affiliations:** From the a Department of Ocular Pathology, King Khaled Eye Specialist Hospital, Riyadh, Saudi Arabia; b Department of Ophthalmology, King Fahad University Hospital, Al-Khobar, Saudi Arabia; c Department of Ophthalmology, Prince Salman Hospital, Riyadh, Saudi Arabia

## Abstract

**BACKGROUND AND OBJECTIVE:**

Conjunctival nevi are benign lesions with wide variation in clinical and histopathological features. The differentiation between benign nevi and other pigmented lesions is essential. The aim of our study was to identify the distribution of the histopathologic types of conjunctival nevi among the Saudi population and to provide the basic knowledge needed for proper clinical diagnosis.

**PATIENTS AND METHODS:**

This retrospective study of surgically excised benign conjunctival nevi was conducted at a tertiary care eye hospital from 1995 to 2006. Clinical data was collected from medical records and the histopathologic features reviewed by a single pathologist.

**RESULTS:**

A total 105 conjunctival nevi were included from 104 consecutive patients (mean age, 26 years, 54 males and 50 females). The anatomical location was the bulbar conjunctiva in 83%, juxtalimbal in 12%, caruncle in 4% and palpebral in 1%. The lesion was removed for cosmetic reasons in 38% while 8% of the lesions were removed to rule out malignancy. The compound nevus was the commonest (72%) in all age groups, followed by subepithelial nevus (24%) and finally junctional nevus (3%).

**CONCLUSIONS:**

The distribution of the histopathologic types of this tumor in our population matches the pattern in other areas of the world with the compound nevus being the commonest lesion. However, fewer lesions among our patients are removed to rule out malignancy.

Melanocytic lesions of the conjunctiva have diverse clinical and histopathologic features. They can be classified into nevi (congenital, which are present at birth or appearing within the first 6 months of life, and acquired), melanosis (congenital and acquired), intermediate melanocytic proliferations and malignant melanoma. The three types of melanocytes involved in these lesions are “dendritic,” which are most commonly found in benign congenital and acquired conjunctival melanosis, “nevus cells” found in subepithelial, junctional and compound nevi, and “fusiform cells,” which are most commonly found in melanosis oculi and the nevus of Ota.^1^ Most conjunctival nevi appear later in childhood, puberty or early adulthood, and thus are considered acquired.^2^

In dermatopathology, the term “nevus” is used to describe a variety of hamartomatous and/or neoplastic lesions. The term melanocytic nevus is generally considered a benign neoplastic proliferation of melanocytes.^3^ The three most common types of conjunctival nevi are junctional, compound and subepithelial with a phase of growth followed by maturational self-arrest and a stationary phase. Therefore, these entities may represent different stages in maturation and proliferation of melanocytes with early junctional activity and further descent of the nevus cells into the substantia propria.^1^,^2^ The differentiation between these benign nevi and other pigmented lesions is essential to determine appropriate management.^2^

The distribution of conjunctival pigmented lesions in our population is not known. The aim of this study was to provide basic information about the clinical presentation, the histopathologic main features and the frequency of the different types of conjunctival nevi among Saudis.

## PATIENTS AND METHODS

The study was conducted in a tertiary care eye hospital that serves Saudi Arabia. The study was approved by the research department. All pigmented conjunctival lesions surgically excised from Saudi patients during a 12-year period (1995-2006) were histologically reviewed. A total of 105 conjunctival nevi from 104 patients were included. The histopathologic type of the nevus and other features including inflammation, pigmentation and cystic changes were determined by a single pathologist. Relevant clinical data, including the age of the patients at the time of surgical excision, the sex, clinical diagnosis, location of the lesion, indication for surgery and any associated ophthalmic inflammatory condition naming vernal keratoconjunctivitis or simple allergic conjunctivitis, was collected from patient charts.

## RESULTS

The histopathologic diagnosis of a conjunctival nevus was confirmed in 105 lesions from 104 patients. One 22-year old patient had bilateral compound conjunctival nevi. The 104 patients were further classified according to age into 3 groups: pediatric from age 0 to 14 years, adolescence from 15 to 19 years, and adult from 20 to 74 years. There was almost equal distribution according to sex, with 54 males and 50 females. The mean age of the study group was 26 years (range, 1-74 years, standard deviation, 21 years). The location of the nevi was bulbar in 83%, juxtalimbal in 12%, at the caruncle in 4% and palpebral in 1% of lesions. The clinical diagnosis was benign in 79%, suspected malignancy in 8%, and not stated in the remaining 13% of lesions. Almost all the lesions (98%) were clinically pigmented and 18% showed a feeder vessel or prominent vascularization. Recent growth of the nevus was noted in 23% of the cases.

**Figure 1:**
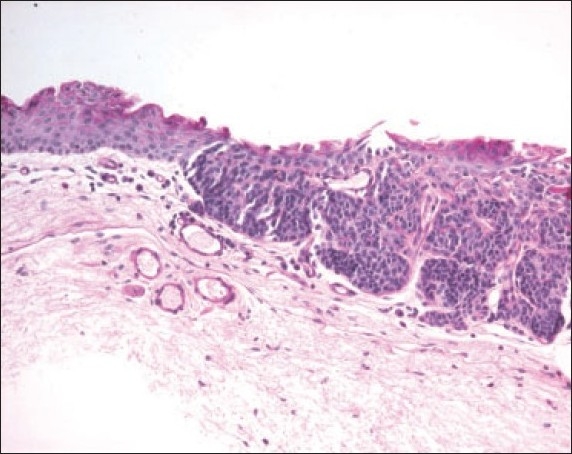
Histopathologic appearance of a junctional nevus in a 19-year-old girl. (Periodic acid schiff ×200).

The histopathologic diagnosis of the 105 excised lesions included compound nevus in 72% (n=76), subepithelial nevus in 24% (n=25) and junctional nevus in 3% (n=3). These three cases of junctional nevi involved a 19-year-old (Figure 1) and 2 children (Figures 2a and 2b). One case of a blue nevus at the caruncle was diagnosed in an adult (Figure 3). The distribution of the histopathologic type of the nevus by age is shown in Figure 4. The compound nevus was the commonest in all age groups and no junctional nevi were diagnosed in adults. Three nevi located at the caruncle were all subepithelial (Figure 5) and one was a blue nevus as described above. Significant pigmentation was noted in 86% (n=90) of the nevi, and 60% (n=63) of the nevi were cystic. The cystic component of the conjunctival nevi is presented in Figure 6. Seventy-one percent (54/76) of compound nevi and 36% (9/25) of subepithelial nevi were cystic.

**Figure 2a:**
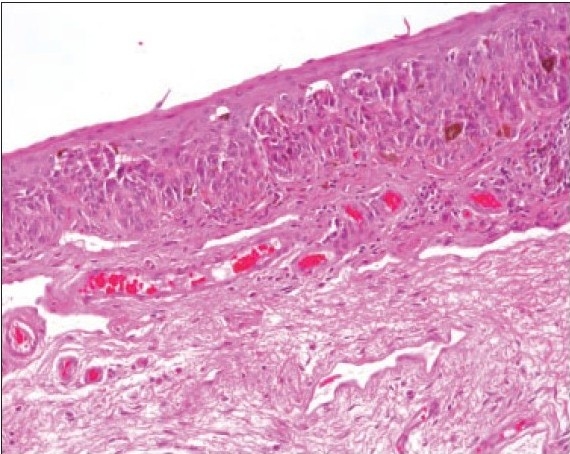
The histopathologic appearance of a junctional nevus in an 8 year old boy. (hematoxylin and eosin ×200).

**Figure 2b:**
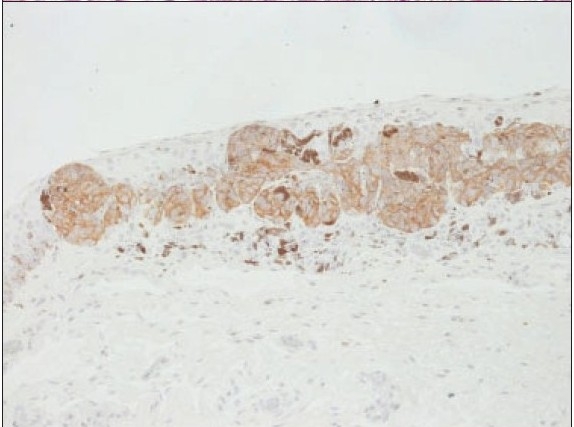
The demonstration of the nevus cells by Melan-A stain ×200.

**Figure 3:**
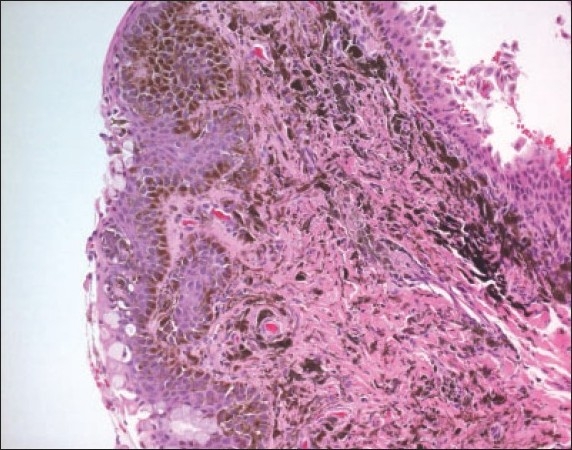
Histopathologic appearance of a blue nevus with proliferating stromal melanocytes. (hematoxylin and eosin ×200).

**Figure 4:**
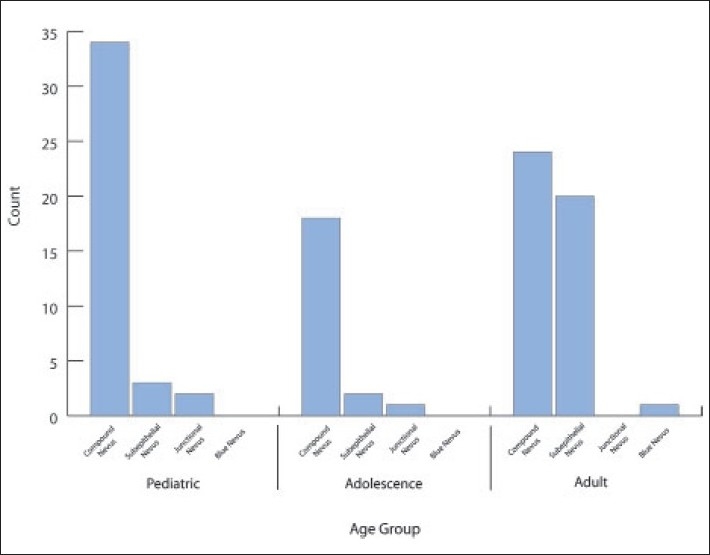
Distribution of the hispathologic types of 105 nevi according to age groups.

**Figure 5:**
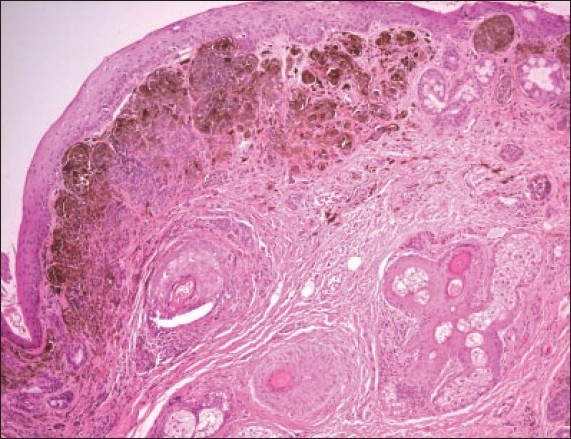
Example of subepithelial nevus at the caruncle in a 14-year-old-boy (hematoxylin and eosin ×200).

**Figure 6:**
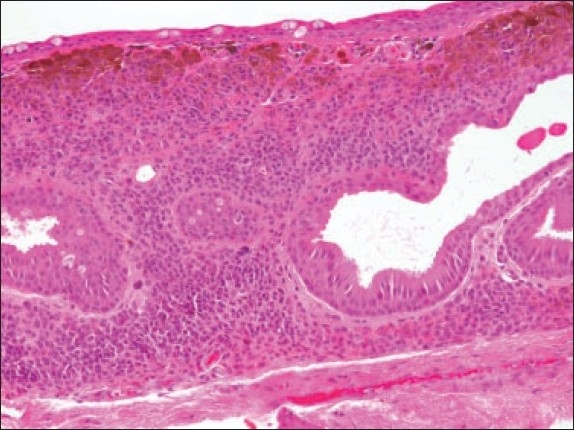
An example of a subepithelial cystic nevus (hematoxylin and eosin ×200).

The commonest indication for surgery was cosmetic in 38% of the lesions, no specific indication in 33%, recent growth or increase in size in 21% and suspicion of malignancy due to the clinical appearance (raised lesion or presence of feeding vessel) and/or change in color in 8%. The location of the eight suspicious lesions, the age, and the final histopathologic diagnosis in these patients are summarized in Table 1. All the suspicious lesions except two were found to be compound nevi. These remaining two cases were subepithelial. The clinical and the corresponding histopathologic features were available for cases 2, 5, 6 and 8, which are presented in Figures 7a, 7b, 8a, 8b, 9a, 9b, 10a and 10b. In the pediatric and adolescence age groups, 48% were inflamed and these were all compound nevi. Of these cases, two patients only had vernal keratoconjunctivitis and a third patient had allergic conjunctivitis. One of these cases is shown in Figure 11a and 11b.

**Table 1:** Suspected malignant lesions age distribution, location and their diagnosis with indication of the presence or absence of inflammation.

Lesion	Age (years)	Location	Histopathologic diagnosis/inflammation
1	7	Juxtalimbal	Compound/moderate
2	7	Juxtalimbal	Compound/none
3	9	Juxtalimbal	Compound/moderate
4	16	Bulbar	Compound/mild
5	17	Bulbar	Compound/moderate
6	21	Juxtalimbal	Compound/mild
7	60	Bulbar	Subepithelial/none
8	74	Caruncle	Subepithelial/mild

**Figure 7a:**
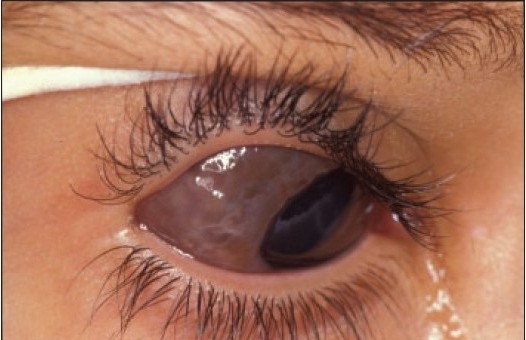
The clinical appearance of a large juxtalimbal nevus of the right eye in a 7-year-old (case 2, Table 1).

**Figure 7b:**
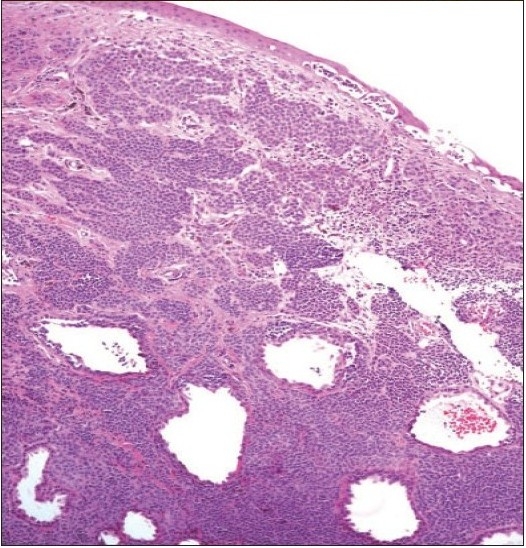
The histopathologic appearance of the compound nevus in the same patient (hematoxylin and eosin ×100).

**Figure 8a:**
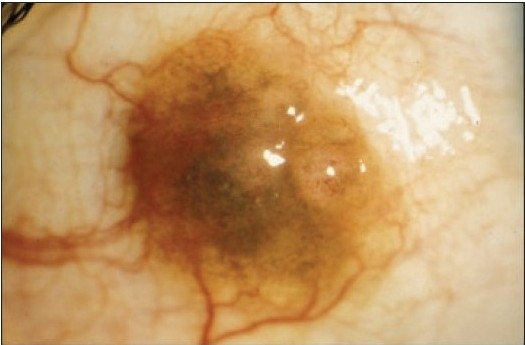
The clinical appearance of a raised bulbar nevus with feeder vessels in the right eye (case 5, Table 1).

**Figure 8b:**
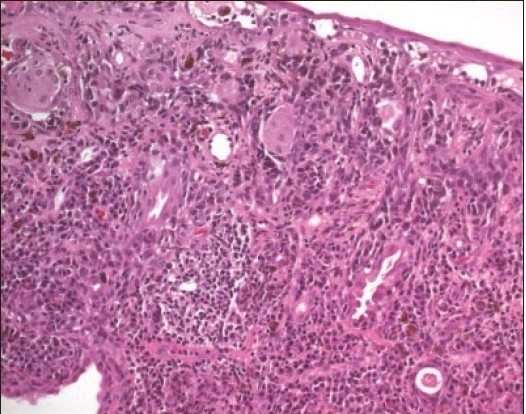
The histopathologic appearance of this compound nevus (hematoxylin and eosin ×200).

**Figure 9a:**
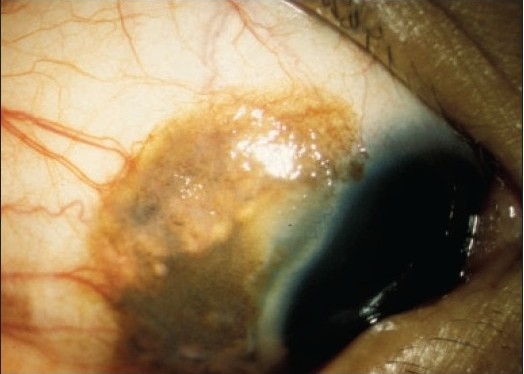
A clinically suspicious juxtalimbal nevus in a 21-year old patient (case 6, Table 1).

**Figure 9b:**
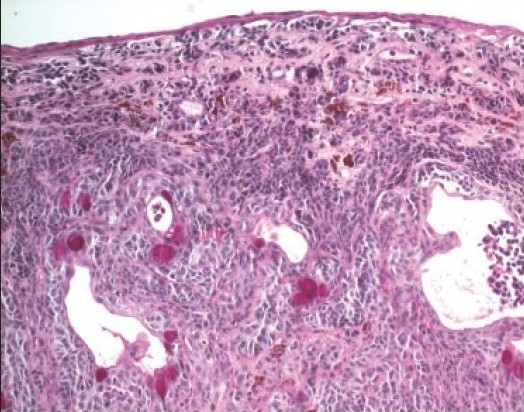
Large compound nevus of this patient with cystic areas. (Periodic Acid Schiff ×200).

**Figure 10a:**
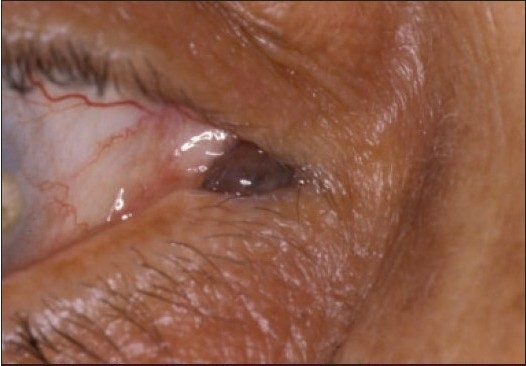
A darkly pigmented lesion at the caruncle of a 74-year-old patient removed to rule out a malignant lesion (case 8, Table 1).

**Figure 10b:**
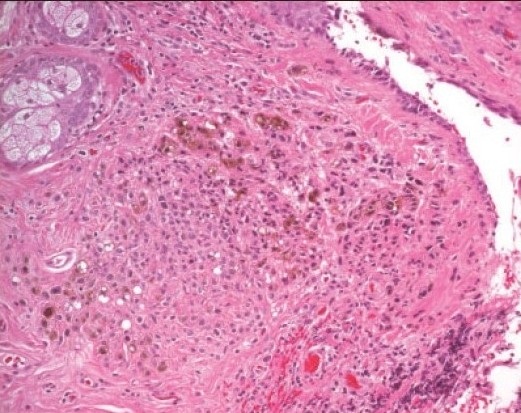
The histopathologic appearance of the caruncular subepithelial nevus in this patient (hematoxylin and eosin ×200).

**Figure 11a:**
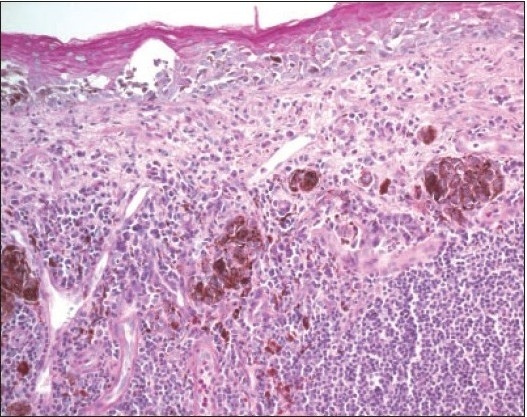
A compound inflamed juvenile conjunctival nevus (IJCN) in association with vernal keratoconjucntivitis (Periodic Acid Schiff ×200).

**Figure 11b:**
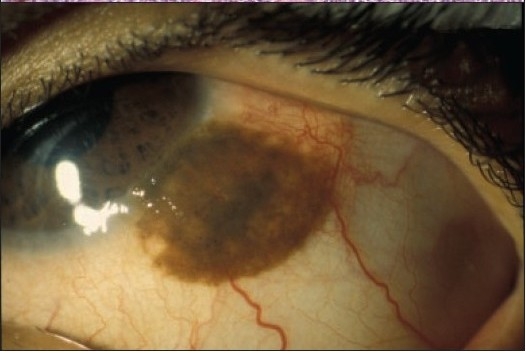
The clinical appearance of this compound nevus in an 18-year-old male.

## DISCUSSION

Melanocytic tumors have been always interesting to many clinicians and pathologists. The ones involving the conjunctiva are more challenging with the evolving modification of their classification.^1^ Benign conjunctival lesions of melanocytic origin can be divided into two main groups: nevi and other benign disorders such as racial pigmentation, post-inflammatory melanosis, primary acquired melanosis and pigmentation related to systemic conditions.^2^ Special attention has been paid to the identification and classification of primary acquired melanosis lesions, which have the potential for malignant transformation. The differentiation between such lesions and benign junctional nevi is essential.^2^

Our results are comparable to a large comprehensive study on conjunctival nevi in 410 consecutive patients by Shields et al.^4^ The age in our patients ranged from 1 to 74 years with the majority of the patients (58%) in the pediatric and adolescent age groups. The mean age at the time of surgical excision was 26 years, which is slightly younger than the mean age of 32 years at initial manifestation in their study.^4^ The correct clinical diagnosis of a nevus was made in 78% of the lesions, while 8% were suspected to be malignant and will be discussed in detail below.

The nevi were most commonly bulbar in 83% followed by juxtalimbal in 12%, at the caruncle in 4% and palpebral in 1%. Gerner et al in their study of 343 conjunctival nevi in Denmark described bulbar lesions in 33%, caruncle in 29%, limbal in 27% and at the eyelid margin in 1%.^5^ Shields et al also described a different site distribution with 72% bulbar, including the ones at or behind the limbus, 15% at the caruncle and 11% at the plica semilunaris.^4^ However, they described the rare occurrence of conjunctival nevi in the tarsal conjunctiva (1%) or the fornix (1%). It has been suggested that the presence of a nevus in the palpebral and forniceal region should raise the suspicion of malignancy and early biopsy.^4^,^5^ We had one palpebral lesion only in a 70-year-old, which was histopathologically proved to be a benign subepithelial nevus. Bulbar conjunctival nevi generally stop abruptly at the limbus and typically do not involve the corneal epithelium or stroma. Such an invasion would be unusual.^1^,^4^ Shields et al^4^ specifically looked at this feature and described rare involvement of the cornea in <1%. None of our juxtalimbal lesions showed corneal involvement.

Table 2 compares our results with Shields et al.^4^ There was a statistically significant difference in history of enlargement of the lesion prior to surgery, pigmentation of the lesion by clinical examination, and indication for surgery. Our histopathologic distribution of the types of nevi was quite similar to other series.^4^,^6^ The commonest was compound nevus in 72%, followed by subepithelial nevus in 24%, junctional nevus in 3% and blue nevus in 1%. We found that compound nevi are the commonest in all age groups. Our three cases of junctional nevi were in the pediatric and adolescence age groups. This confirms the conclusion by Shields et al that compound and junctional nevi are usually found in younger aged group, while subepithelial and blue nevi are usually found in slightly older aged group.^4^ Blue nevus cells presumably originate from the incompletely migrated melanocytes of the substantia propria and are typically located deep in the nevocellular component. Although malignant cellular blue nevi have been diagnosed elsewhere, none have been reported in the conjunctiva.^2^ The only case of blue nevus in our series was in a 22-year-old male with a brown lesion at the caruncle and no prominent vascularization or cystic appearance. The nevus was removed for cosmetic reasons. Shields et al also demonstrated absence of cysts and feeder vessels in their four cases of blue nevi.^4^ Similar findings were reported by McDonnel.^7^

**Table 2:** Comparison between the results of our study and those of Shields et al.^4^

Parameter	Current study (% of total=105 lesions)		Shields et al. (% of total=410 lesions)		*P*
History of enlargement of the lesion prior to surgery	24 (23%)		176 (43%)		<.0001^a^
Most common site of lesion	Bulbar		Bulbar		-
Pigmentation of the lesion by clinical examination	103 (98%)		344 (84%)		<.0001^a^
Indication for surgery	Cosmetic	40 (38%)	Cosmetic	16 (4%)	<.0001^a^
	Recent growth	22 (21%)	Recent growth	32 (8%)	
	To rule out malignancy	8 (8%)	To rule out malignancy	95 (23%)	
	Others	35 (33%)	Others	267 (65%)		
Most common histopathologic diagnosis	Compound nevus		Compound nevus		-

a Significant by Chi-square test.

Zamir at al studied 63 inflamed juvenile conjunctival nevi (IJCN) in patients younger than 20 years.^8^ Seventy-five percent of their patients had a history of allergic disease. They suggested an association of this unique entity with allergic conjunctivitis.^8^ Some recent data also suggest that this association is due to the modulation of eosinophil properties by lesional fibroblasts partly through nerve growth factor.^9^ However, when we analyzed our patients in the pediatric and adolescence age groups (under the age of 20 years) 48% of the compound nevi where classified as IJCN and 12% (3/25) had documented vernal or allergic eye disease.

In conclusion, our findings demonstrate that the distribution of conjunctival nevi in our population is similar to other studies with compound nevus being the most common diagnosis in all age groups and bulbar lesions being the most common site. However, in our population, almost all nevi were clinically pigmented and the most common indication for surgery was cosmetic concerns. Further studies of IJCN and other conjunctival pigmented lesions including primary acquired melanosis and malignant melanoma are recommended.
